# What is in a look? The accountability of gaze in trajectories to conflict

**DOI:** 10.3389/fpsyg.2024.1436191

**Published:** 2024-10-28

**Authors:** Rebecca Clift

**Affiliations:** Department of Language and Linguistics, University of Essex, Colchester, United Kingdom

**Keywords:** gaze, conflict, staring, accountability, other-initiated repair, presequences, conversation analysis

## Abstract

This study investigates the role of gaze in initiating episodes of conflict by examining, using multimodal conversation analysis, a set of cases in which a recipient is prompted to speak by another’s extended gaze. In these cases, this recipient response may be, e.g., “What,” or a more elaborate demand for an account, such as “Why are you looking at me like that for?” Here we investigate the characteristics of the gaze that prompts such responses, and what actions such responses constitute. While “What” compositionally resembles other-initiated repair, its sequential position characterizes it as a so-called “go-ahead” action. In these cases, the sequential positioning of such gazes, constituting it structurally as a so-called “pre,” alongside its durational characteristics and facial expression, are examined to identify the normative associations of gaze and subsequent conduct that make such gazes accountable.

## 1 Introduction

From Darwin (1872) onwards, there has been a recognition that a particular form of fixed or studied eye-gaze—a so-called “stare”—in specific contexts may be associated with hostility.^1^ At its extreme, such hostility is perhaps most acutely embodied in the phenomenon of the so-called “hate stare” leveled at African-Americans by White Americans. The white journalist, John Howard Griffin, passing as an African American man in the segregationist south of the US in the 1950s, captures one such occurrence:

It came from a middle-aged, heavy-set, well-dressed white man. He sat a few yards away, fixing his eyes on me. Nothing can describe the withering horror of this. You feel lost, sick at heart before such unmasked hatred, not so much because it threatens you as because it shows humans in such an inhuman light. You see a kind of insanity, something so obscene the very obscenity of it (rather than its threat) terrifies you (Griffin, 1961).

Griffin’s account leaves no doubt as to the status of such gazes as actions. In addition, on a more mundane level, perceiving oneself to be stared at in a public space is one that many will have experienced. Robert De Niro’s famous line from “Taxi Driver”—“You lookin’ at me?”—captures the response to the perceived threat conveyed by an extended eye gaze. Such instances testify to the culturally salient associations between extended gaze and potential hostility, an association picked up by Goffman, who, on observing brief moments between strangers in public spaces, anchors this hostility in the “invasion of informational preserve” (Goffman, 1971, p. 54) that staring represents.

What follows contrasts with Goffman’s observations of interactions between strangers in public by examining cases in domestic settings in which a party responds verbally to an extended gaze by another, who is an intimate—in our cases, a family member. In so doing, we aim to establish the interactional implications of the studied gaze and the ways in which the hostility apparent when it is deployed between strangers is played out in its use between intimates.

The data here are taken from corpora of filmed family interaction. Most were taken from a corpus, edited parts of which were originally broadcast on Channel 4 (UK) from 2008 to 2009 as part of the TV series “The Family.” The broadcast programs were taken from approximately 1,500 tapes of two British families filmed continuously in their homes across 100 days by over 20 cameras. One family can be seen in extracts 2, 4, 8, 9, 10, and 11; the other can be seen in extract 1. Although the whole corpus comprised a whole week’s worth of raw unedited footage of one family, as well as the broadcast footage of both families, all the extracts transcribed here are taken from the broadcast footage.^2^ Extract 7 was taken from a corpus of informal family interactions recorded by the participants themselves.^3^

**Figure 1:**
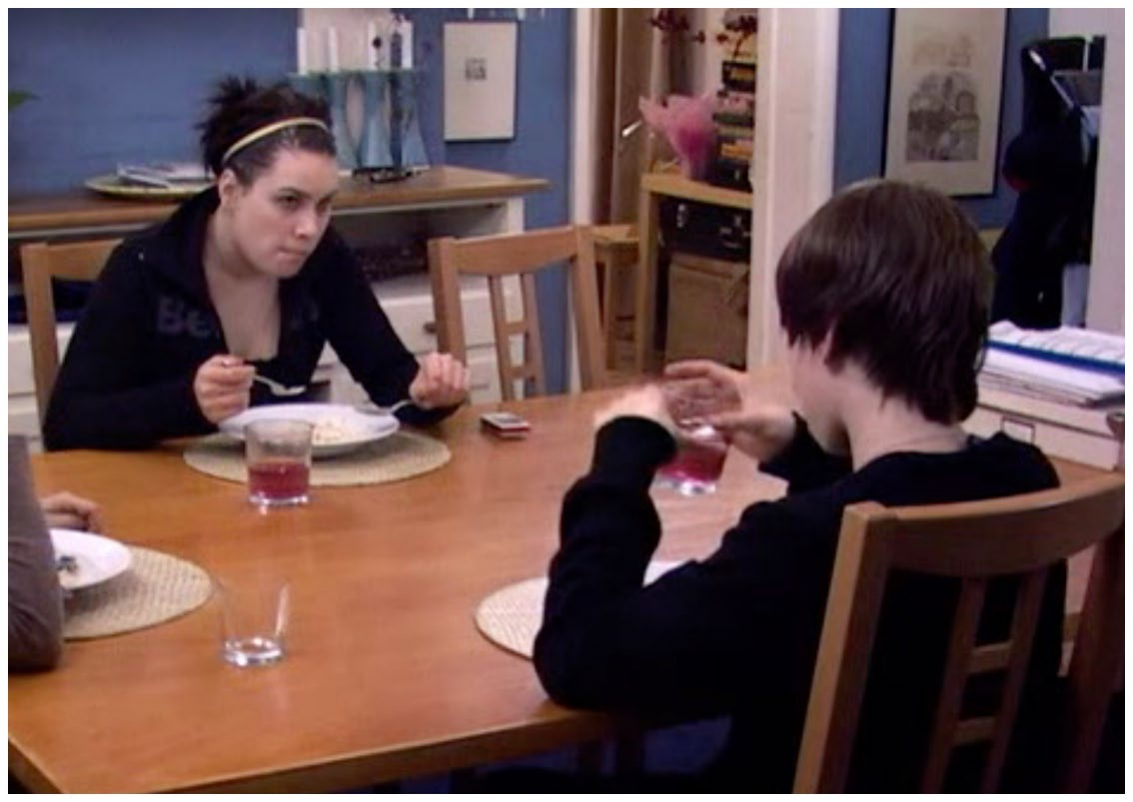
Emily’s studied gaze at Tom, extract 2, l.5. © Dragonfly Film and TV Productions Ltd. Reproduced with thanks to Dragonfly Productions.

Figure 1 shows one instance of the phenomenon of interest. It captures the studied gaze of over 3 s by a teenage girl, Emily, across the dining table to her younger brother, Tom—an action that initiates a trajectory leading Emily to abandon her meal and go to her bedroom in the midst of the ongoing interactional conflict.

The focus in such cases is how such gazes are treated interactionally by both parties—that is, their sequential implications. As we shall see, it is overwhelmingly the case that such gazes, such as the one above, are taken to adumbrate some kind of negative or even hostile stance and initiate a trajectory of interactional conflict.

Of course, experience tells us that in other contexts (such as between lovers or intimates generally, for example), the studied mutual gaze may also be deployed to more affiliative ends. We return to such alternative possibilities in due course, but in the corpus of hundreds of hours’ worth of filmed family interaction examined for this research, it was the case that *none* of the instances of studied eye-gaze that we encountered were in such highly affiliative environments.^4^ This empirical skewing, reflected in the data examined here, suggests a strong preference for the studied gaze to be taken as potentially hostile. We examine this phenomenon in what follows. We initially sketch relevant work on gaze and embodiment, before discussing an initial instance of a held gaze as clearly problem-implicative. In examining a number of cases in analytic detail to track the interactional trajectory from the gaze initiation onwards, we then investigate what action the response to the gaze constitutes. This, in turn, illuminates the action that the held gaze itself is implementing. We show how the fixed gaze is taken to adumbrate a problem, before going on to examine some cases where this problem-implicativeness and potential source of conflict may be defused. In conclusion, we also discuss a clear exception to the hostility implied by a fixed gaze.

## 2 Background: work on gaze and embodiment

Over 60 years of psychological research has sought to elaborate on Darwin’s original observations on gaze, from Gibson and Pick (1963), Argyle and Dean (1965), and Kendon (1967) onward; for a broad overview, see Hessels (2020). The affective possibilities of eye-gaze have been a consistent focus in such research (see, e.g., Green et al., 2003; Eisenbarth and Alpers, 2011; and for a review, see Hietanen, 2018). Work in multimodal Conversation Analysis (CA) has, since Goodwin (1979, 1981), sought to build on this study by bringing a consideration of sequential positioning (on which, see, e.g., Clift et al., 2013) to the study of gaze and embodiment in interaction. Unlike much previous work on gaze which focuses on establishing the “meaning” of particular forms of gaze, CA examines actions and the practices that deliver actions across sequences, and uniquely takes the participants’ systematic responses to those practices to be criterial for understanding a practice as displaying, for example, hostility or tenderness.

In a pioneering CA study of children from 1 to 2½ years of age, Kidwell (2005) shows how while engaging in activities such as biting, pushing, or hitting, children can look to their caregivers and differentiate between a “mere look” from them and a more extended, sanctioning gaze. What Kidwell calls “the look” is of relatively long duration, alights on a target or targets, and is produced as “an activity in its own right” (Kidwell, 2005, p. 429). These children are thus analyzing their own conduct: doing something sanctionable, then looking to see the ways in which they are being monitored. A central feature of “the look” is that the gaze is held. In this respect it resembles a number of other embodied phenomena characterized by so-called “holds”: body posture (Sikveland and Ogden, 2012; Groeber and Pochon-Berger, 2013; Li, 2014; Floyd et al., 2016; Manrique, 2016), hand gestures (Clift, 2020), and facial expressions (Clift and Rossi, 2023) can all be used as well as gaze (Rossano, 2012) to indicate the at-that-moment unresolved status of a sequence and disruption to its progressivity (Schegloff, 2007, pp. 14–15)—and, as such, problem-implicative. All of this work has served to emphasize the importance of examining, not just the gaze itself, but its associated embodiment and the sequential environment in which these are produced for understanding its implications for action.

## 3 An initial instance: the held gaze as problem-implicative

**Figure 2:**
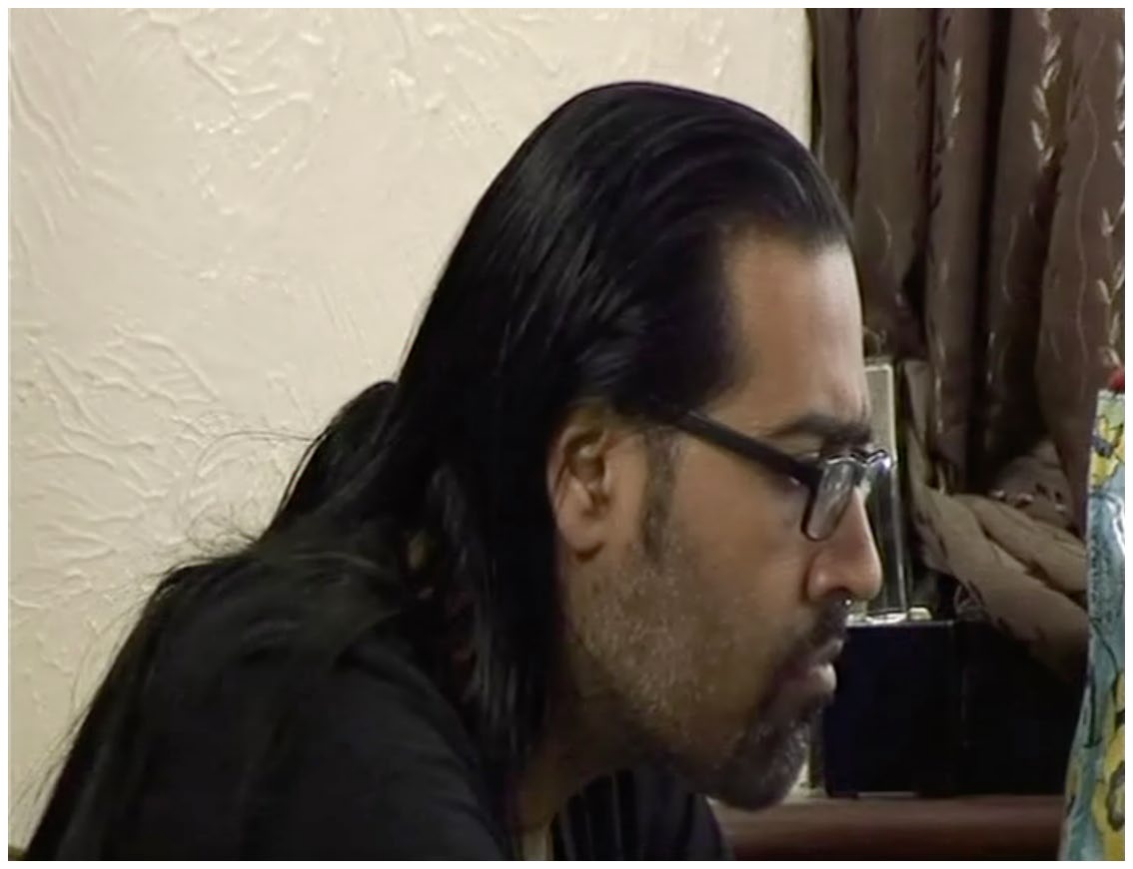
Sunny’s gaze, extract 1, l.4. © Dragonfly Film and TV Productions Ltd. Reproduced with thanks to Dragonfly Productions.

In mundane interaction, the problem-implicativeness of a held gaze is nowhere more apparent than in the following instance. Here a young woman, Shay, voices her distress (l. 1-2), having just phoned her estranged mother, then registers her fiancé, Sunny, holding his gaze (see Figure 2).^5^ She responds, after 3.5 s of a visible gaze from Sunny, with an apology to him (l.5) for what she takes to be the offense she has caused:

(1) Angry^6^ (Clift F:2:3: 41–54)

Sha=Shay (eye gaze *); Sun=Sunny, Shay’s fiancé (eye gaze +; embodiments •); Pol=Polly, Sunny’s mother.
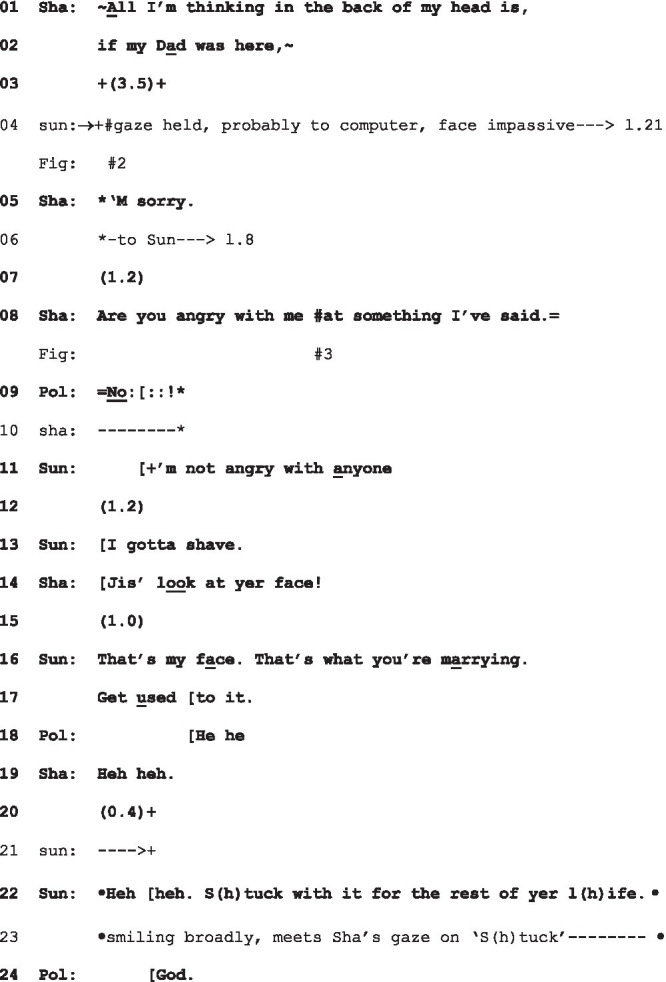


**Figure 3:**
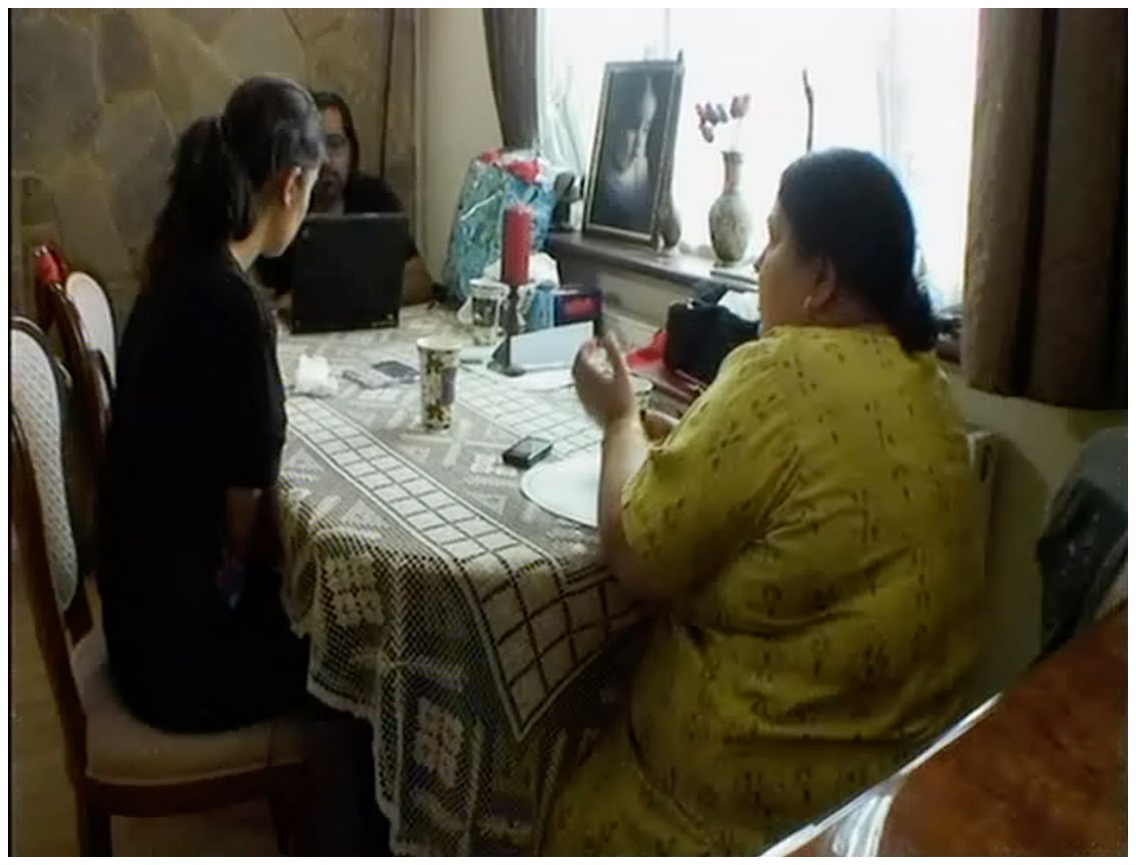
Shay’s gaze to Sunny, extract 1, l.8. © Dragonfly Film and TV Productions Ltd. Reproduced with thanks to Dragonfly Productions.

A display of distress might expect some kind of empathic response (see Heritage, 2011, on empathic moments)—particularly in the case of intimates—and Shay looks to Sunny in the wake of her turn (Figure 3). But as we see in Figure 2, Sunny meets her expression of distress with an impassive face and his head in a hold. As Figure 3 shows, he is sitting at a computer, and it is likely that he is focussing on the screen; in any case, he does not respond to Shay. Her apology and, in the face of further silence (l.7), her inquiry as to whether she has caused offense, suggest her understanding of his fixed gaze, impassive expression, and non-response collectively to project hostility: “are you angry with me at something I’ve said” (l.8). It is Poli, Sunny’s mother, who immediately and most straightforwardly produces a prosodically emphatic denial (l.9) followed by Sunny, whose “I’m not angry with anyone” (l.11) constitutes a somewhat tepid rebuttal. Shay’s response to this, “Jis’ look at yer face,” (l.14) provides a retrospective account of her apology and its rationale. It is this response—and its reference to “yer face”—that suggests Shay understands the held gaze, with the impassive face, to be projecting negativity—and specifically anger. Sunny’s delivery of his subsequent response (ll.16–17), delivered in a brusque, mock-angry tone, plays on this misapprehension.

This exemplar thus makes plain the problem-implicativeness, if not the potential hostility, attached to a held gaze with an impassive face, although of course this instance was not directed to a target and was produced in a sequential position where some response might have been expectably due. In each of the cases that follow, we further explore the sequential implicativeness of the sustained gaze by examining instances which, in contrast, are directed at a specific target and which initiate a verbal sequence.

## 4 The gaze as action

The following instance, captured in Figure 1, takes place in the course of a family dinner. In the wake of an exchange between Jane, the mother, and her 14-year-old son, Tom, about his long fringe (l.1–3), 19-year-old Emily looks up from her meal and, with an impassive face, produces a sustained gaze of 4 s at Tom. In the course of the gaze, she suspends her cutlery in a hold so that it is evident that she is wholly preoccupied with looking; the “activity in its own right” observed by Kidwell (2005, p. 429). In response to the look, Tom, who was in the course of bringing a glass to his lips, halts the raise, tilts his head up to fix his gaze on Emily, and says: “What,” l.7:

(2) Nothing

Jan=Jane, mother (gaze €, embodiments ‡); Emi=Emily, 19 year-old daughter (gaze *, embodiments •); Tom=Tom, 14 year-old son (gaze +, embodiments %)
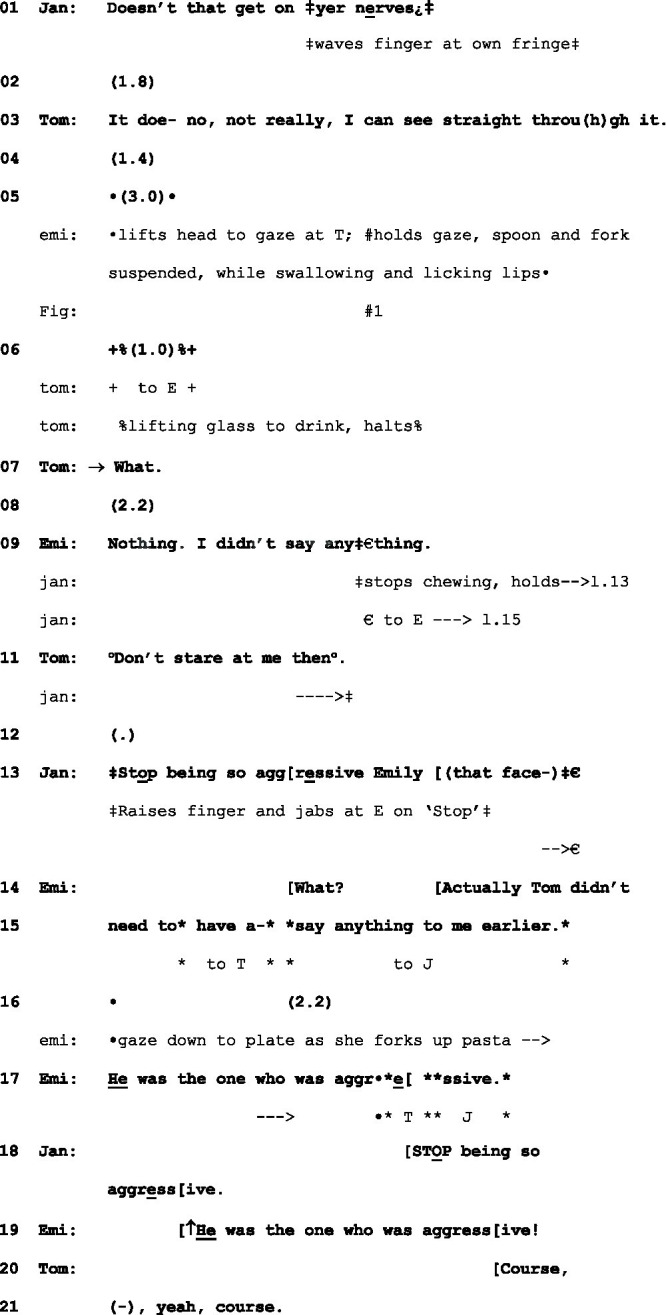


Emily’s response (l.9), with its brusque denial that she said anything, itself receives a rebuke from Tom explicitly referencing the stare (l.11) and thus accounting for his verbal initiation of the sequence in l.7. It is at this point that Jane intervenes; her assessment of Emily’s behavior as “so aggressive” (l.13) clearly treats Emily as the antagonist in this exchange—and one that leads, some turns later, to Emily abandoning her meal and going up to her bedroom. However, lls. 14-15 show that Emily’s response treats an earlier exchange as the origin of the conflict, with Tom as the initiator. The hostility attributed to the direct gaze in this exchange, and subsequently embodied verbally by Emily, in contrast to that in extract (1), thus turns out to be warrantable—and initiates an episode of conflict in the here-and-now.

In attempting to identify the kind of action a studied gaze is taken to be implementing, the treatment by Emily of Tom’s “What” is here critical. In insisting that she “did not say anything” (l.9)—something which is, of course, true—she treats it as an other-initiated repair. Her own subsequent “What?” at l.14 might appear, at first glance, to warrant this treatment of Tom’s turn, as it initiates repair on his softly produced l.11.

Instances such as the one below might also appear to endorse an understanding of “What” as initiating repair. Here, Michael at l.4 initiates repair on Shane’s “some” in l.1, and is completed by “saline solution” at l.6:

(3) Saline solution (Schegloff, 1997, p. 515)

Chicken Dinner, 48:34–49:11. Sha = Shane; Mic = Michael.
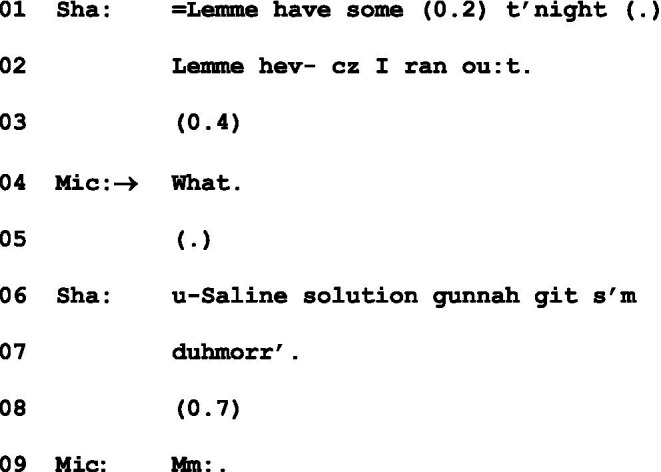


However, as Schegloff et al. (1977) note, the repair is addressed to problems in the understanding of, and initiated on, *talk*—and so Tom’s “What” as a response to Emily’s look cannot be initiating repair. But another instance in which “What” responds to a gaze, and moreover, another such trajectory toward conflict, sheds light on this issue. In (4) below, a recipient—as it happens, Emily—responds to a studied gaze with “What.” Earlier in the evening, Emily had been upbraided by her parents for consistently going out late at night to clubs and then calling in sick to work. The extract below takes place later that evening. Just beforehand, Simon, Emily’s father, having opened the front door to a taxi driver whom Emily has evidently called, then enters Emily’s bedroom, walks silently across the room, then stops and looks at her for 3 s:

(4) What is going on

Sim = Simon, father (gaze +, embodiments %); Emi = Emily, 19-year-old daughter (gaze *, embodiments •).
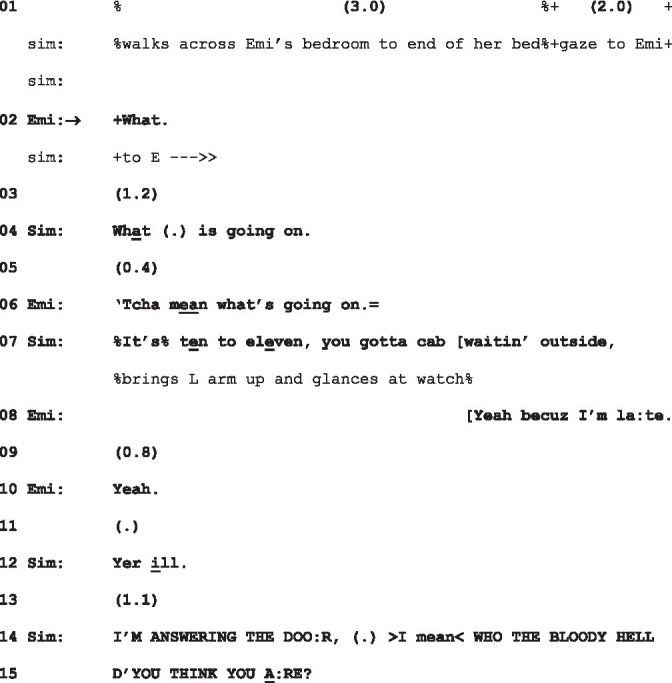


Simon’s (delayed) response to Emily’s “What” here delivers, in l.4, a complaint in the form of a question: “what is going on.” This complaint itself gets repair initiated upon it by Emily and is subsequently elaborated on by Simon. Emily’s defense (l.8), selectively addressing the fact in Simon’s turn concerning the cab, but not the manifest complainable about the time, only prompts Simon to continue listing her offenses (l.12 and l.14) and then produce an assessment in the form of a rhetorical question (l.14-15), so escalating the conflict. In due course, this exchange ends with Emily storming out of the house, leaving her parents in visible distress.

Once again, then, a sustained gaze is responded to by “What”—a trajectory leading ultimately to conflict—but the response to it shows that it is treated distinctly in (2) and (4). What Simon’s response to Emily in (4) shows us is that the sustained gaze he directs at her is in fact the complaints he delivers in l.7, 12, 14 and 15. In other words, he treats Emily’s “What” as a go-ahead (Schegloff, 2007, p. 30) to his complaint. In (5) below, we see similarly a “What” being treated as a go-ahead (at l.2, and then subsequently at l.8) to a turn which is a pre-announcement (“Y’know w’t I did las’night?”, l.1):

(5) A terrible thing

Schegloff (1997), pp. 516–517; Hyl = Hyla; Nan = Nancy.
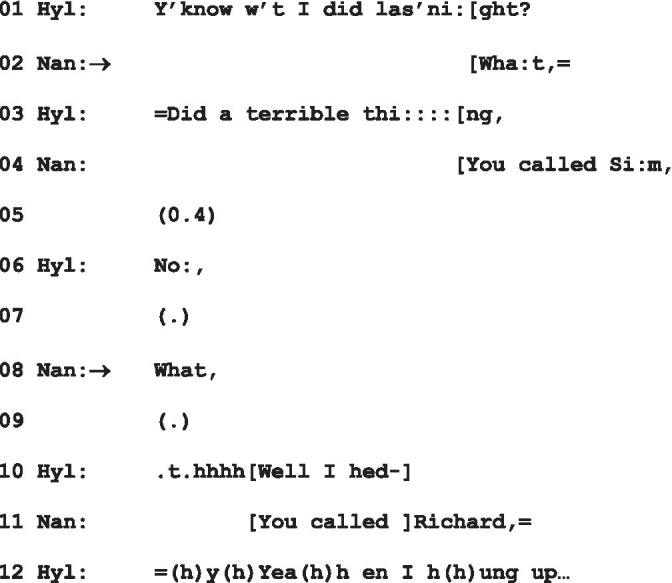


Schegloff further notes that:

Forms of turn-constructional unit that can be used to initiate repair on another’s prior turn can also be used as types of response in what we have come to call “presequences” of various types. The so-called “generic pre-sequence,” which serves advance notice of some upcoming “business” without marking what that is, is the summons/answer sequence (1997, pp. 513–514).

In (6) below, we see one such exemplar. Amidst other ongoing activities, Fred is summoned by his mother (l.2), to which he responds with the aligning go-ahead “What” (l.3):

(6) Salami (Schegloff, 1997, p. 514) Nao = Naomi; Fre = Fred; Ann = Anne, mother.
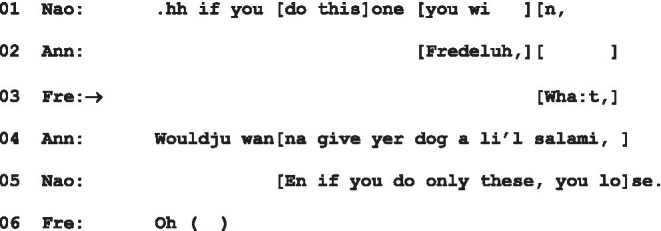


There is thus independent evidence that “What” produced by Tom in (2), notwithstanding Emily’s disingenuous treatment of it as an other-initiated repair, is clearly a “go-ahead” to the prefacing action constituted by the sustained gaze—as indeed is that produced by Emily herself in (4). In structural terms, then, the look is a so-called “pre,” serving, in Schegloff’s words, as “advance notice of some upcoming business” (Schegloff, 1997, p. 514). However, we have already seen enough evidence to suggest, *contra* Schegloff, that unlike the summons, which may be heard as equivocal with respect to what it prefaces, as a “pre” the look is not necessarily treated as a neutral action. Shay’s response to Sunny’s fixed gaze (for all that it is not directed at her) and the responses in extracts (2) and (4) suggest a technical preference for understanding the look to adumbrate some kind of challenging or problematic action for the recipient—an understanding that is indeed borne out by the interactions that follow.

## 5 Fixed gazes as adumbrating a problem

The origins of the fixed gaze as adumbrating a problem can be identified in the turn-taking system and specifically the practices for selecting the next speaker. Both Goodwin (1980) and Lerner (2003) discuss gaze as one resource for selecting next speaker; as Lerner notes: “It is common for speakers to look at or look for an addressed recipient as they begin to speak, and for the onset of a speaking turn to occasion a reciprocal gaze by coparticipants to determine if they (alone) are being addressed” (2003, p. 180; see also Goffman, 1963 and Cary, 1978 for observations on gaze as initiating encounters). The common retort “Don’t look at me” responds to the implication, carried by a gaze, that the gaze producer is about to prevail upon the gaze recipient to act in some way.^7^ Such an implication aligns with the norms of progressivity:

‘…Moving from some element to a hearably-next-one with nothing intervening is the embodiment of, and the measure of, progressivity…’ (Schegloff, 2007, p. 15)

So, when a gaze producer does not produce a “hearably-next” action, stalling progressivity, and, in its stead, fixing the gaze on a recipient with an impassive expression, the gaze becomes, at the very least, accountable. In the following, this accountability rises to the surface of the talk following a fixed gaze of 0.8 s (l.11):

(7) Panera Drive-Thru

(25_FCSp14_AT_: 26:30 Family in the kitchen 1 Mandelbaum, Rutgers)

Dau = Daughter (* eye gaze*, $embodiments$); Mom, (+eye gaze+, &embodiments&).

The family is cooking together, with Mom and Daughter making up a packet of taco sauce. Dau is at the hob stirring the sauce while Mom takes the packets out of the cupboard to hand to Dau.
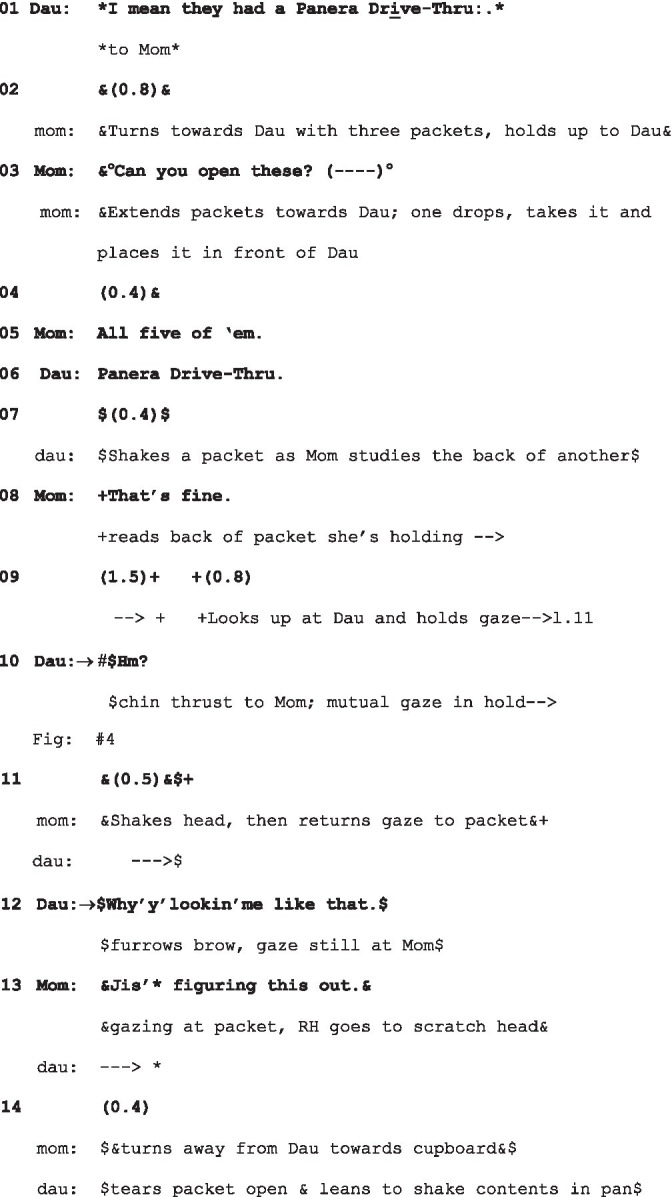


**Figure 4:**
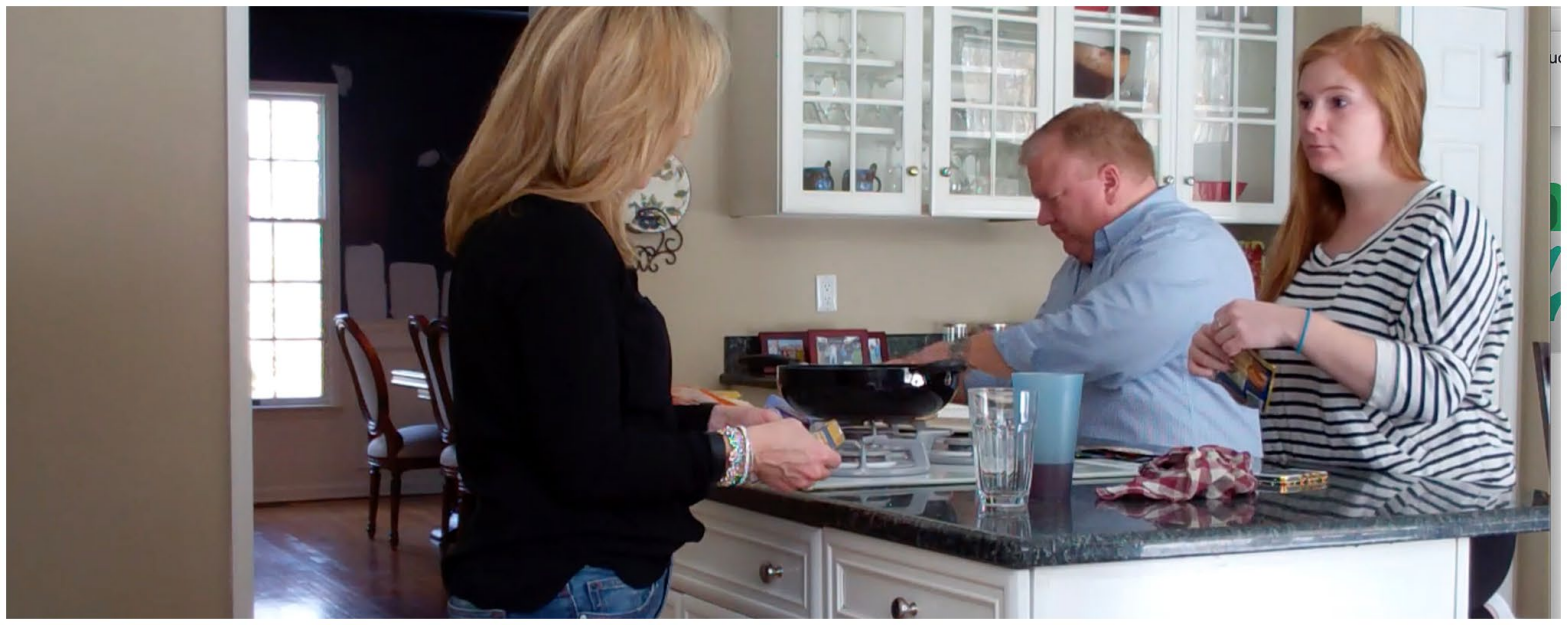
Mom’s gaze to daughter and daughter’s “Hm?,” extract 7, l.10.

Just as in extract (2) and indeed (4), the response to the look, here “Hm?,” deploys a form commonly used for other-initiated repair, but which is here a go-ahead (see Figure 4).

But before Mom responds, Daughter follows up with a challenging question: “Why’y’lookin’me like that” (l.12), putting on record the accountability of the fixed gaze. Mom’s response, unlike those in (2) and (4), defuses the potential hostility in its account: “Jis’ figuring this out” (l.13).

In some cases, the go-ahead is omitted altogether, and the gaze producer is called directly to account. In the following, Jane enters the bedroom in the wake of a row with Emily and stands still for 5.8 s, looking at Simon as he finishes his phone call. He turns around to see her gazing fixedly at him:

(8) What you looking at me like that for

Sim = Simon (*eye gaze*, +embodiments+); Jan = Jane (%eye gaze%, $embodiments$).
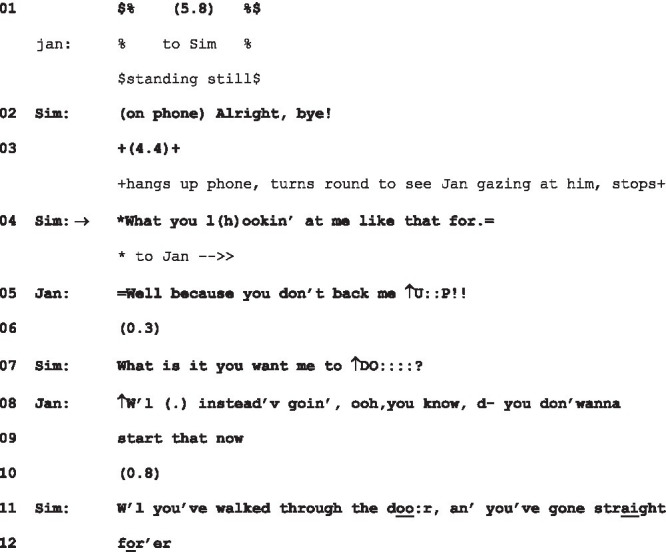


Simon’s “What you l(h)ookin’ at me like that for” (l.4)—itself a hearably upgraded format from the responsive “why” used in (7)—similarly calls the gaze-producer to account, “like that” explicitly pointing to a way of looking that is being challenged. Here, its infiltrated laugh token indicates the delicacy of the challenge (see, e.g., Clift, 2012 on laughter marking delicacy) and gets an immediate, latched response from Jane: a complaint with some articulatory force and prosodic animation “Well because you do not back me U::P!!” (l.5). Simon’s response—another challenging question—almost exactly mimics Jane’s prosody and articulation, and thus escalates the conflict.

A more escalated exchange still, following a fixed gaze, can be seen in the following. As in the previous extract, the gaze is treated as accountable—and, as in the previous extract, the format “what…for” is used. However, the gaze itself is produced in an environment that is already fissile. Emily has been summoned to the living room by her parents to address her recent wayward behavior. She arrives and crosses the room, watched by her parents, bounces down on the sofa, clears her throat, sniffs (all actions that might be hearable as preparing to speak), but then fiddles with a blanket, and then lifts her gaze to fix it on her mother:

(9) Filthy Looks

Jan = Jane; Emi = Emily (gaze *, embodiments •); Sim = Simon (gaze +, embodiments %).
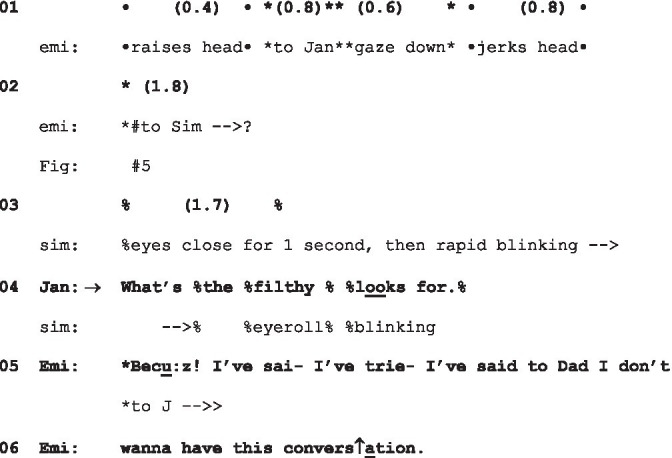


**Figure 5:**
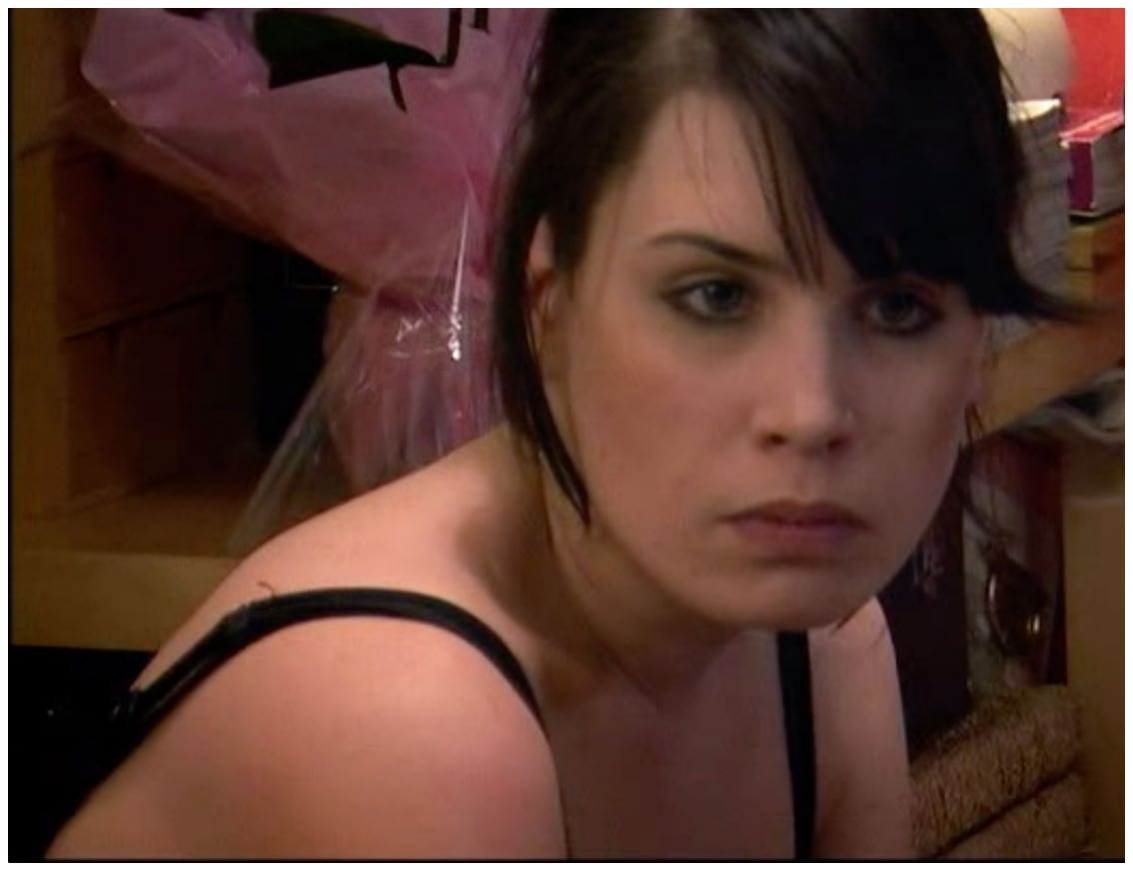
Emily’s gaze at extract 9, lls.1–3. © Dragonfly Film and TV Productions Ltd. Reproduced with thanks to Dragonfly Productions.

From the time Emily sits down to Jane’s response is a whole 12 s, and her gaze from when she lifts her head to gaze at her parents, over 6 s (see Figure 5)—all while her parents sit silently watching her. Jane’s response to this gaze is, as in (8), a challenging question—but, while in (8) Simon’s assessment of Jane’s look was not made lexically explicit, here Jane’s assessment of the look is made plain. The reference to “filthy looks” constitutes a double upgrade of the familiar phrase “dirty look”: lexically, from “dirty” to “filthy,” and morphologically, from one such “look” to several. Emily’s response does not challenge either the question or the assessment it embodies. Her emphatic production of “Becu:z!” as its own TCU initially resists producing a further account, but an account does then follow, albeit with some dysfluency attending its launch—and one that grounds the hostility of the look in her reluctance to engage—one that is reported, in an epistemic upgrade (Clift, 2007), as having been registered with her father earlier: “I’ve said to Dad I do not wanna have this conversation” (ll.5–6). What subsequently follows is a highly antagonistic exchange.

We have thus seen a number of instances in which a fixed gaze is taken to be adumbrating some kind of problem or challenge. In the most benign context (7), we see a gaze being called to account; the subsequent response works both to account for the look and to reassure, and conflict is thereby averted. In (8) and (9), the fixed gaze initiates a conflict sequence, just as had (2) and (4). However, as we have seen, these sequences themselves all follow from prior episodes of conflict – either immediately prior, as in (4), (8), and (9), or, as Emily makes clear in (2), some time previously: “Tom did not…need to…say anything to me earlier” (lls. 14–15). So, in these cases, the fixed gazes can be seen as the initiating action in a potential renewal of hostilities.

This is not to suggest, of course, that all fixed gazes initiate conflict sequences. Extract (7), where there had been no prior antagonism between the parties, shows a gaze addressed and accounted for unproblematically. It is clear, then, that in some contexts a fixed gaze may be managed to more peaceable outcomes.

## 6 Defusing hostility

While the majority of instances in our corpus, as represented in the cases mentioned above, showed gazes initiating a trajectory of conflict, we only found two cases involving fixed gazes where such conflict was averted, one by the gaze-producer, and one by the gaze-recipient; in different ways, they throw into relief the characteristics of the conflict-initiating gaze. The first is a case in which the gaze is accompanied by (lighthearted) verbal indications of trouble, and so in that respect distinct from the cases examined so far. Here, Simon sits having breakfast with Emily and Tom. He gazes over the table at Emily, who spent the previous night at a nightclub, “Icon,” and whose eye-makeup is visibly smeared:

(10) Black eyes F1:2 3:52–4:06

Sim = Simon (*gaze* + embodiments+); Emi = Emily ($gaze$ %embodiments%).
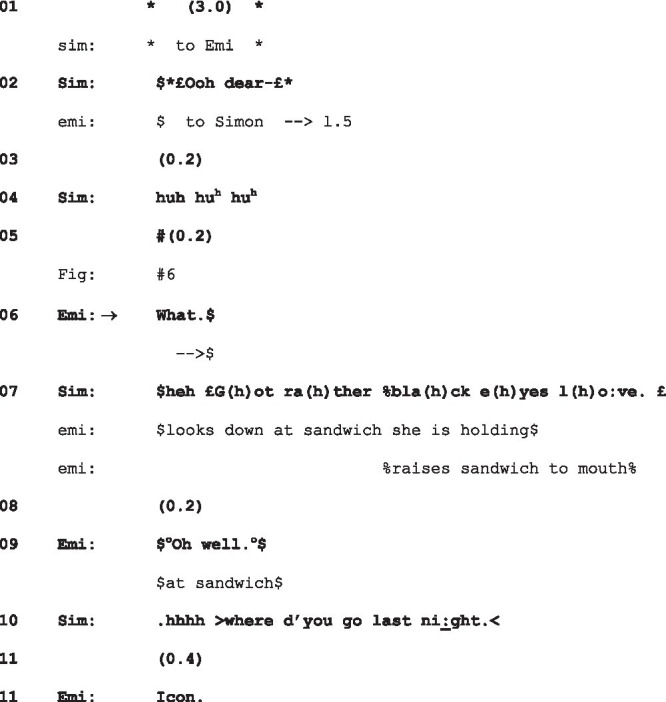


**Figure 6:**
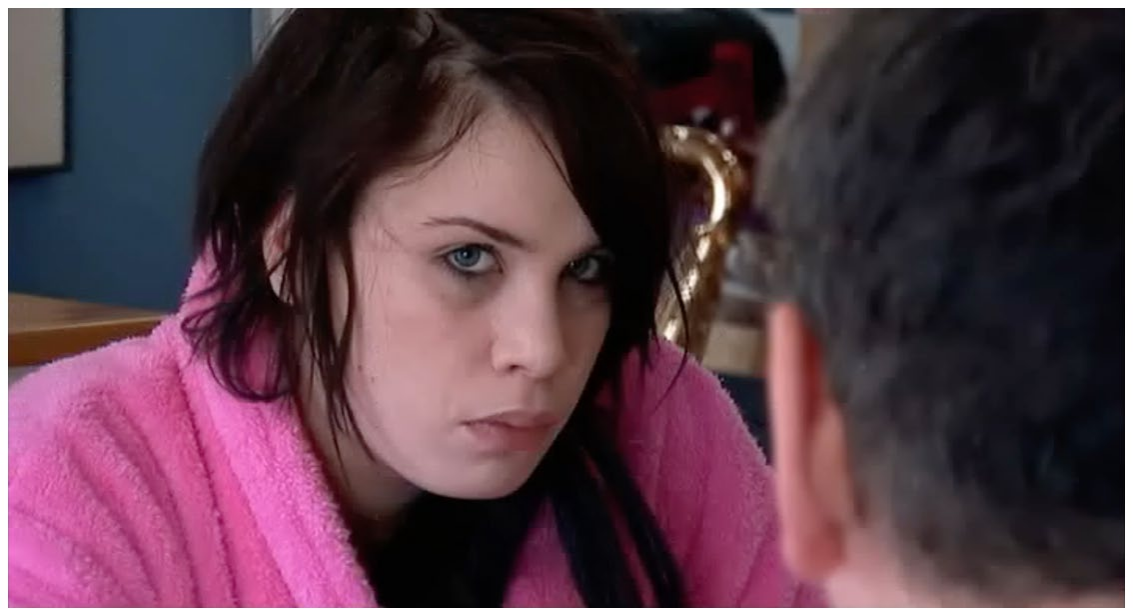
Emily’s response to Simon’s gaze, just before “What” (l.5), extract 10, l.4. © Dragonfly Film and TV Productions Ltd. Reproduced with thanks to Dragonfly Productions.

Simon’s fixed gaze is thus accompanied by his verbal trouble-alert, “Ooh dear” and then a laugh, as he looks at Emily. Her go-ahead “What” (l.6) in response is produced as she in turn gazes at Simon with visible wariness (Figure 6); notwithstanding Simon’s jocular overtures, Emily declines to join him in his laughter, hearable as it is as a potential tease, laughing, not with, but at her.

Simon’s response to the go-ahead, “£G(h)ot r(h)ather bla(h)ck e(h)yes l(h)ove£” (l.7)—a negative assessment accounting for his prior trouble-alert and laughter, is itself laughter-filtrated. At this, Emily, looking at her bacon sandwich, lifts to her mouth, saying “Oh well”—a display of resignation that registers Simon’s assessment without taking issue with it. Simon’s highly redressive action in producing his gaze underscores the fact, demonstrated by extracts (2), (4), (7), (8), and (9), that it is the fixed and silent gaze that is taken to be potentially hostile.

**Figure 7:**
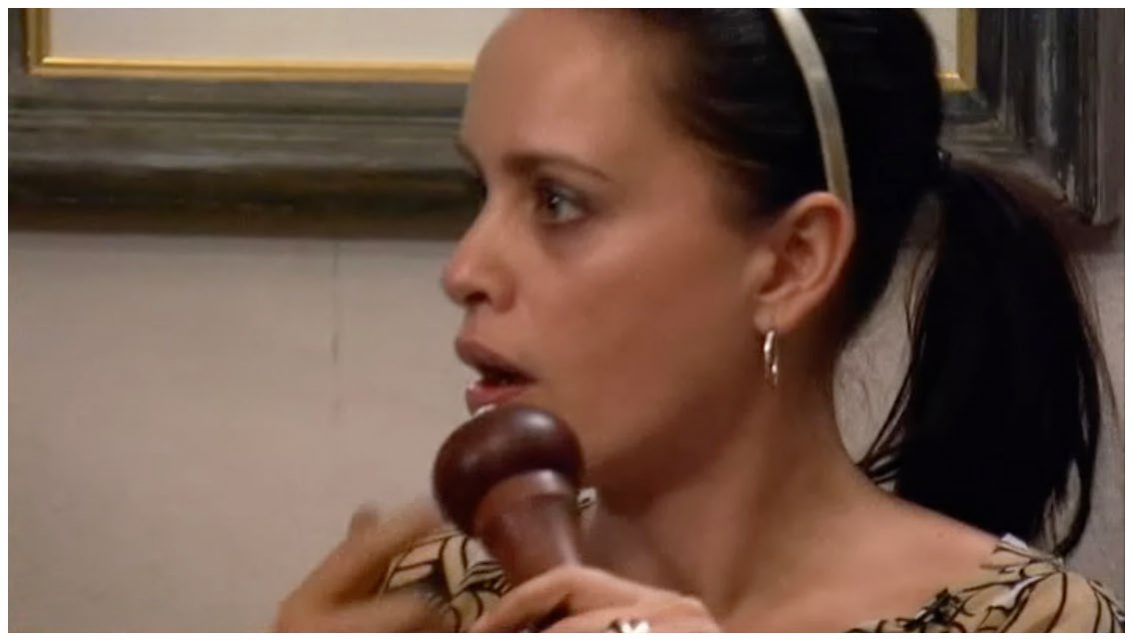
Jane points to pepper mill, extract 11, l.2. © Dragonfly Film and TV Productions Ltd. Reproduced with thanks to Dragonfly Productions.

The other instance where a gaze did not escalate into conflict is one where the recipient figures what the gaze is adumbrating, and takes measures to defuse it. The context is a highly affiliative one—a Valentine’s meal, prepared by Simon for Jane. As she prepares to eat, she compliments the paella in front of her (l.1), but as she raises the pepper mill, visibly about to grind pepper on her food, she sees Simon gazing at her. She lifts the pepper mill, points at, and, with a straight face (Figure 7), produces a defense to what she thereby implies is about to be his complaint: “Tasted it first”:

(11) Tasted it first

Jan = Jane (*gaze* + embodiment+) Sim = Simon (%gaze% &embodiment&).
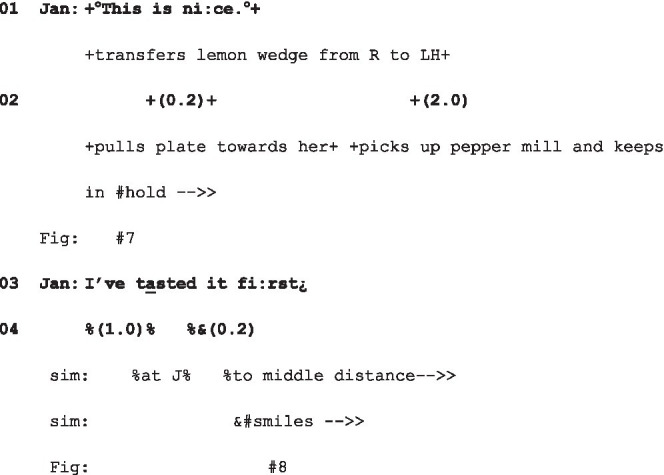


**Figure 8:**
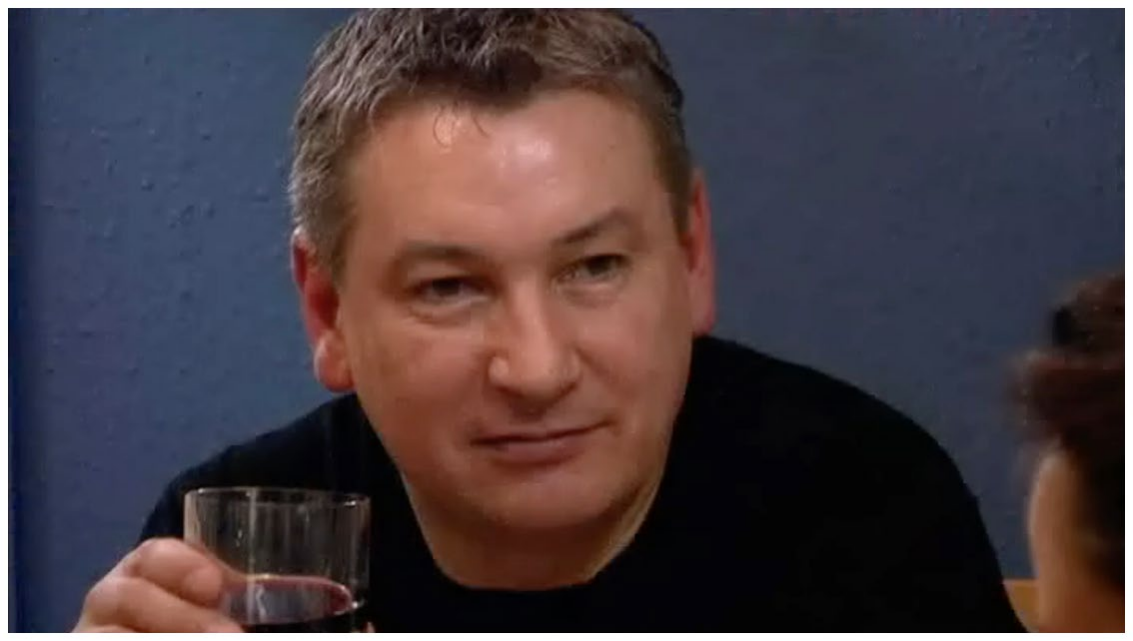
Simon’s response to Jane, extract 11, l.4. © Dragonfly Film and TV Productions Ltd. Reproduced with thanks to Dragonfly Productions.

Simon, in response, produces a wry smile (Figure 8).

While reminiscent of Kidwell’s (2005) sanctionable gazes by caregivers, the distinction is that here the adult interactants share a history, and one that is likely to have been drawn on here by Jane. “I’ve tasted it first” (l.3) invokes a history of exchanges where Simon has cause to complain that his cooking is being seasoned before having been tasted. Jane’s response thus pre-empts this adumbrated complaint, her retort deftly defusing the nascent hostility.

In this connection, it is perhaps unsurprising that the dining table should figure in a number of the instances [(1), (2), (10), and (11)] here. As a site for a (standardly) daily gathering, opportunities for monitoring—and so potentially sanctioning—others’ behavior are both ample and recurrent, facilitated by the positions of the participants in Kendon’s F-formation, either in so-called vis-à-vis or L-arrangements (Kendon, 1990, p. 209).

Although these last two instances show fixed eye gazes initiating a trajectory that does not lead to conflict, the fact that, in both cases, redressive action to avert conflict needs to be taken—in (10) by the gaze-producer, and in (11) by its recipient—itself constitutes evidence of the conflict-initiating potential of such gazes.

## 7 Conclusion

**Figure 9:**
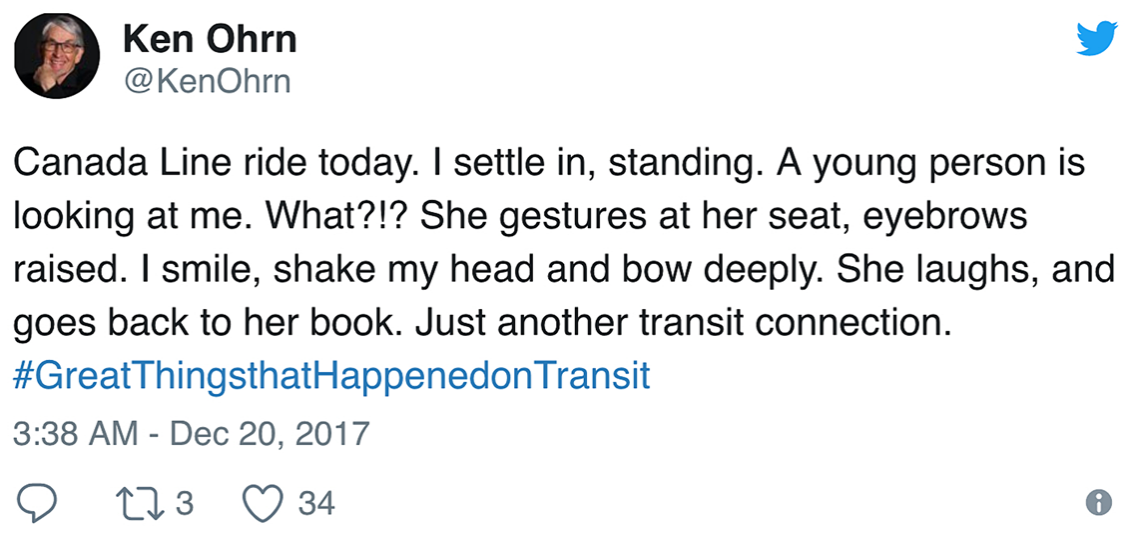
Twitter, 20th December 2017, Transit Line Connection - What?!?” via Twitter @KenOhrn.

The potential hostility attached to a studied, silent gaze in public spaces among strangers was found to be overwhelmingly replicated in our data, recorded in domestic settings among intimates. Structurally, this fixed gaze is a so-called “pre” action, designed to adumbrate some business—and that, overwhelmingly, the nature of that business is taken to be challenging in some form. In such cases, the designed eye gaze initiates an interactional trajectory toward conflict. A few cases we have shown constitute exceptions to this usual trajectory from studied eye gaze to conflict. The first instance, in (1), showed a fixed gaze mistakenly taken to be hostile. Extract (7) showed a gaze being treated with wariness, and being accounted for by its producer. In both, there was clearly an orientation to the projection of a potential negative stance, neither of which turned out to be so designed. Extract (10) was a gaze which was, like that in (7), treated warily but mitigated with verbal resources and laughter and so conflict was headed off. Moreover, extract (11) also shows conflict averted by the gaze-recipient with an account for the action taken to be the target of the projected complaint. There were no instances in the data of a fixed and silent gaze designed to be preliminary to an unequivocally affiliative action. The single instance of the latter identified in the course of this study was one, not recorded, but reported on social media—and not between intimates, but strangers. In the wake of Elon Musk’s reported complaints in 2017 about traveling by public transport, several responded on what was then Twitter with stories about the great things that had happened when they had taken busses and trains, such as meeting their spouses or best friends. The following relates one such encounter, initiated with a studied gaze—the reportability of an affiliative moment on public transport underscored here by the wordlessness^8^ of the exchange (Figure 9).

As reported here, the studied gaze from a stranger prompts a (from the lack of quotation marks, silent) go-ahead “What”—the punctuation suggesting not so much aggression but bemusement—the response to which is an embodied offer, then graciously declined. That this reported instance was the only one identified in which a fixed gaze is designed to adumbrate a wholly positive action further attests to its relative rarity, at least in the data of English interaction.^9^

This empirical skewing—moreover, on the data of intimates, not strangers—thus suggests an overwhelming preference for taking the fixed gaze to be projecting an upcoming negatively polarized action. This observation dovetails with the preference for progressivity and the corresponding association of bodily holds with at-that-moment unresolved issues or problems in the talk. The recipient of such a gaze, as we have seen, can then produce a responsive go-ahead (such as “What” or “Hm?”) which maintains a relatively neutral stance with respect to what is likely to come, or an immediately more confrontational one, demanding an account for the characteristics of the look (e.g., “Why you looking at me like that for”/“What’s the filthy looks for”). Such actions may be initiating a verbal course of action, but of course have their source prior to the talk, in the fixed gaze. In this respect, the sequences launched by the gaze are structurally what Schegloff (2007) calls “retro-sequences,” invoking a source-outcome relationship, in which, he notes:

…the first recognizable sign that such a sequence is in progress generally displays that there was “a source” for it in what preceded, and often locates what that source was. But note that the source engendered nothing observable—indeed, was not recognizable as “a source”—until the later utterance/action, billing itself as an “outcome,” retroactively marks it as such. Their “firstness” follows their outcome, though their occurrence preceded it. These are sequences *launched* from their *second* position (2007, p. 217).

Locating the source involves monitoring what in the environment might be called to account (hence, as noted earlier, the dining table as a particularly rich site for possible candidates, where one might take another to be judging them for, e.g., slurping food or licking a knife); Shay’s query to Sunny, “Are you angry with me over something I’ve said” in (1) exactly captures this uncertainty. In this respect, Eckert’s concept of an “indexical field” as a set of possible interpretations that undergo indexical specification *in situ* (Eckert, 2008, p. 454; see also Heritage, 2016, p. 209) is a useful one. A studied gaze evokes an indexical field of possibilities, narrowed down by both compositional features (such as facial expression) and sequential context. The resonance of this with Garfinkel’s (1967, p. 34) characterization of the indexical and reflexive properties of language and action is clear.

The instances we have examined, in the data of mundane domestic interaction, thus show very clearly how one practice—the fixed gaze—can in fact initiate an interactional trajectory that may be increasingly conflictual. Schegloff, contemplating the extreme outcomes to which such trajectories may ultimately lead, observes that understanding how such horrors arise is the first step in attempting to address them:

Rape, abuse, battering, etc., do not exist in some other world, or in some special sector of this world. They are intricated into the texture of everyday life for those who live with them. How else are we to understand their explosive emergence where they happen if not by examining ordinary interaction with tools appropriate to it, and seeing how they can lead to such outcomes…how else—when confronted by the record of singular episodes—are we to understand their genesis and course, how else try to understand what unwilling participants can do to manage that course to safer outcomes, how else try to understand how others might intervene to detoxify those settings? (Schegloff 1999, pp. 561–562).

In examining the sequential implications of a particular kind of look, it has thus been possible, as a first step in such an endeavor, to illuminate the normative assumptions of accountability and progressivity that underlie the resources we deploy in pursuing courses of action.

## Data Availability

The original contributions presented in the study are included in the article/supplementary material; further inquiries can be directed to the corresponding author.

## Footnotes

^1^This article was written while I was in receipt of a Distinguished Scholar Award from the Harry Frank Guggenheim Foundation for the project “Behind Closed Doors: Trajectories to Violence in Intimate Interaction,” and I gratefully acknowledge the support of the Foundation, and of the Arts and Humanities Impact Acceleration Fund at the University of Essex in undertaking this research. I have shown the data in various presentations, among them, at Teacher’s College, Columbia University, New York, Language and Social Interaction working group, in 2018; at Huddersfield University in 2019; at Uppsala University, for the Transition and Sustainability of Communicative Competencies in Interactions Involving Young People with Communication Disabilities (TRANSCOMM) Workshop 2: Tensions, Troubles and Displays of Affect, in 2020; Ocean University, Qingdao, Shandong, China, and the Dutch Linguistic Society, Dutch Society for Applied Linguistics and LOT, Netherlands Graduate School, in 2021; and the University of Salerno in 2022. I am grateful to the audiences at these institutions, and the reviewers and editors of this article, for their constructive comments and feedback.

^2^The edited nature of the footage clearly raises issues for the analyst, but I hope it will be apparent that, as far as can be established, none of the extracts presented here have been analytically compromised.

^3^I thank Jenny Mandelbaum for making these data available to me. I am also grateful to Dragonfly Productions for making “The Family” data—both edited for broadcast, and raw, unedited data—available.

^4^This of course may be an artifact of the kinds of contexts video-recorded for conversation-analytic research (on which, see Clift, 2024).

^5^Gazes are timed from the beginning of the gaze to the recipient’s response. I am grateful to Dr. Nicole Smith for helping me with the timings of the gazes here in ELAN.

^6^The transcriptions conform to the conventions developed by Lorenza Mondada and are set out at https://www.lorenzamondada.net/_files/ugd/ba0dbb_3978d2a34cf44376adb7a341975d23aa.pdf.

^7^This is embodied in reports of episodes where a mutual gaze prefaces particular actions—in the following newspaper headline, an astonished response to an exceptional occurrence: “‘We just looked at each other and started laughing’: Raynes Park mum defies odds of ‘one in 200 million’ to give birth to identical triplets” *Your Local Guardian*, 20th September 2016.

^8^The silent nature of the exchange is not the focus here, but it is possible that this may be fitted to the ambient noise on public transport.

^9^A referee for this study contests the relative rarity of such positive actions compared to negative ones as adumbrated by a fixed gaze. This may be attributable to cultural variation. In my experience (and thus anecdotally), I have observed greater tolerance for the fixed gaze—and thus greater likelihood that it adumbrates some positive action, such as a compliment—in some cultures than others; hence, the caveat that the findings are observable only for English.
